# Immunosuppressive Activity of 8-Gingerol on Immune Responses in Mice

**DOI:** 10.3390/molecules16032636

**Published:** 2011-03-22

**Authors:** Jing Lu, Shuang Guan, Xue Shen, Wenhui Qian, Guoren Huang, Xuming Deng, Guanghong Xie

**Affiliations:** 1 Department of Veterinary Pharmacology, College of Animal Science and Veterinary Medicine, Jilin University, Changchun, Jilin 130062, China; E-Mails: xlujing1@yahoo.com.cn (J.L.); gshuang1973@126.com (S.G.); 2 Laboratory of Nutrition and Function Food, Jilin University, Changchun, Jilin 130062, China; E-Mails: shenqingyansmile@126.com (X.S.); jtqwh@163.com (W.Q.); 249843949@qq.com (G.H.)

**Keywords:** immunosuppression, 8-gingerol, humoral immune, cellular immune

## Abstract

8-Gingerol is one of the principal components of ginger, which is widely used in China and elsewhere as a food, spice and herb. It shows immunosuppressive activity on the immune responses to ovalbumin (OVA) in mice. In the present study, we found that 8-gingerol suppressed lipopolysaccharide (LPS) and concanavalin A (ConA)-stimulated splenocyte proliferation *in vitro*. *In vivo*, 8-gingerol not only signiﬁcantly suppressed Con A-, LPS- and OVA-induced splenocyte proliferation (P < 0.05) but also decreased the percentage of CD19^+ ^B cells and CD3^+^ T cell (P < 0.05) at high doses (50, 100 mg/kg). Moreover, OVA-speciﬁc IgG, IgG1 and IgG2b levels in OVA-immunized mice were reduced by 8-gingerol at doses of 50, 100 mg/kg. These results suggest that 8-gingerol could suppress humoral and cellular immune responses in mice. The mechanism might be related to direct inhibition of sensitized T and B lymphocytes.

## 1. Introduction

**Figure 1:**
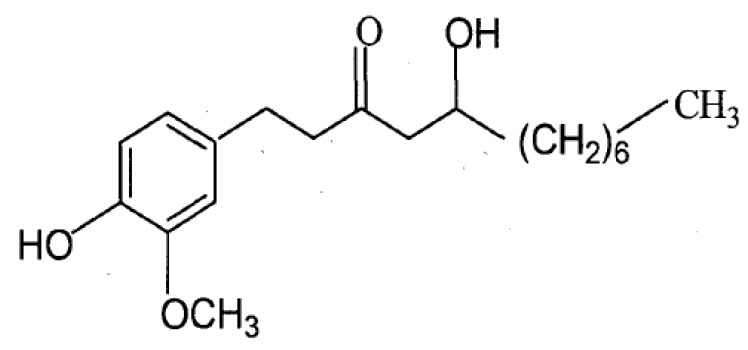
Structure of 8-gingerol.

Today, many diseases, such as systemic lupus erythematosus and rheumatoid arthritis, are attributed to autoimmune diseases. On the other hand, rejection is common in organ transplantation surgery. Curing these diseases depends on suppressing the body’s immune responses. In the clinic numerous immunosuppressive drugs such as cyclosporin A, FK506, rapamycin, cyclophosphamide or prednisone, have now been adopted to use for organ transplantation and to treat some autoimmune diseases. However, these drugs have a narrow therapeutic range [1]. In addition, a number of serious adverse effects including headaches, insomnia, hepatotoxicity, induction of diabetes and so on have been described [2]. As a consequence, it is very necessary to search for new, potential immune-suppressant with improved safety profiles In recent years, suppression of immune responses by medicinal plant products as a possible therapeutic measure has become a subject of scientiﬁc investigation. Several plant components such as triperine and triptolide [3], brazilin [4], sinomenine [5]* etc.*, possess immunsuppressive effects. Ginger, the powdered rhizome of the herb *Zingiber ofﬁcinale*, is widely used as a spice, food and herb throughout the world. In Traditional Chinese Medicine, ginger has been used as a treatment for rheumatism, nervous diseases, gingivitis, toothache, asthma, stroke, constipation and diabetes [6]. 8-Gingerol, as shown in Figure 1, is one of the principal pungent components of ginger [7]. Previous research have proved that 8-gingerol had various pharmacological functions, such as anti-platelet aggregation activities [8,9], spasmolytic activity [10], modulation of macrophage functions [11], inhibiting LPS-induced PGE 2 production and LPS-induced COX-2 expression [12] and 5-HT_3_ receptor blocking activity [13], but the immunoregulatory effects were mainly found in macrophages and the mechanism of 8-gingerol was poorly understood. In this study, we investigated 8-gingerol for its *in vitro* and *in vivo* immunosuppressive activity on the cellular and humoral immune response against OVA in BALB/c mice. 

## 2. Results and Discussion

### 2.1. Splenocyte viability

**Figure 2:**
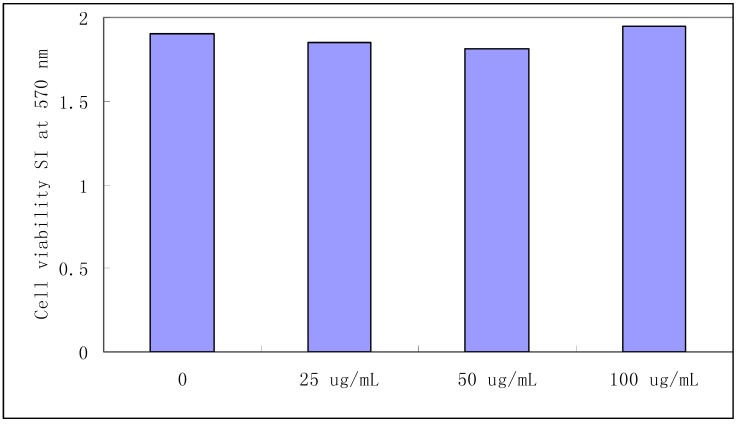
Effect of 8-gingerol on the viability of splenocytes.

8-Gingerol did not affect the viability of splenocytes treated with concentrations ranging from 0 to 100 μg/mL for 24 h, as detected by the MTT assay (Figure 2). It showed no toxic effects on mice splenocytes in concentrations up to 100 μg/mL. These results assured us that the effects of 8-gingerol on splenocytes were due to its immunosuppressive activity, and not to cell death.

### 2.2. Proliferation of splenocytes in vitro

**Figure 3:**
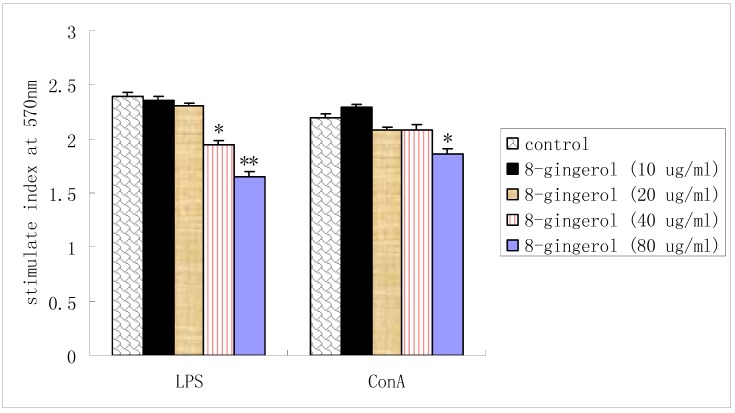
Effect of 8-gingerol on splenocyte proliferation *in vitro*.

LPS-stimulated splenocyte proliferations were reduced by 8-gingerol treatment at doses of 40 μg/mL and 80 μg/mL. Meanwhile, 8-gingerol suppressed ConA-stimulated splenocyte proliferation only at dose 80 μg/mL. There was no signiﬁcant difference at low doses (Figure 3).

### 2.3. Proliferation of splenocytes from OVA-immunized mice

LPS-induced splenocyte proliferation in OVA-immunized mice was signiﬁcantly suppressed by 8-gingerol at doses of 50 and 100 mg/kg. Similarly, when treated with Con A or OVA, the proliferation was also markedly inhibited at dose of 100 mg/kg, but showed little changes at 25 or 50 mg/kg.

### 2.4. Effect of 8-gingerol on splenocyte surface markers in OVA-immunized mice

Treatment of isolated OVA-immunized mouse splenocytes with 8-gingerol resulted in a signiﬁcant dose-dependent downregulation of the CD3^−^ CD19^+^ B cell as determined by membrane expression (Table 1). CD3^+^ CD19^−^ T cell populations were not markedly decreased compared with control, but significantly decreased at 100 mg/kg of 8-gingerol compared with OVA control. The results were from three independent experiments and presented as mean±SD. Statistical difference was accepted at * p < 0.05 or ** p < 0.01.

**Table 1:** Effect of 8-gingerol on CD3+ and CD19+ lymphocytes Subsets in OVA- immunized mice.

Groups	CD3^+^ CD19^-^%	CD3^- ^CD19^+^%	CD3^+^CD19^+^%
**Negative group**	26.17 ± 1.14	27.62 ± 1.25	1.68 ± 0.19
**OVA Control**	32.65 ± 1.22	34.11 ± 1.54	2.51 ± 1.29
**OVA+ 8-gingerol (25 mg/kg)**	33.54 ± 2.05	33.63 ± 1.87	2.85 ± 1.07
**OVA+ 8-gingerol (50 mg/kg)**	31.72 ± 2.06	30.32 ± 1.94*	2.03 ± 0.76
**OVA+ 8-gingerol (100 mg/kg)**	28.65 ± 2.15*	24.8 ± 2.13**	2.66 ± 0.88

### 2.5. Effect of 8-gingerol on OVA-specific serum antibody response

To investigate the effect of 8-gingerol on the humoral immune responses in OVA-immunized mice, serum OVA-speciﬁc antibody levels were measured by ELISA. OVA-speciﬁc IgG levels in the serum were reduced by 8-gingerol at doses of 50, 100 mg/kg compared with OVA control and IgG2b levels decreased at 100 mg/kg. Reduction in serum IgG1 levels was observed in all groups of 8-gingerol -treated mice compared with control. 

### 2.6. Discussion

**Figure 4:**
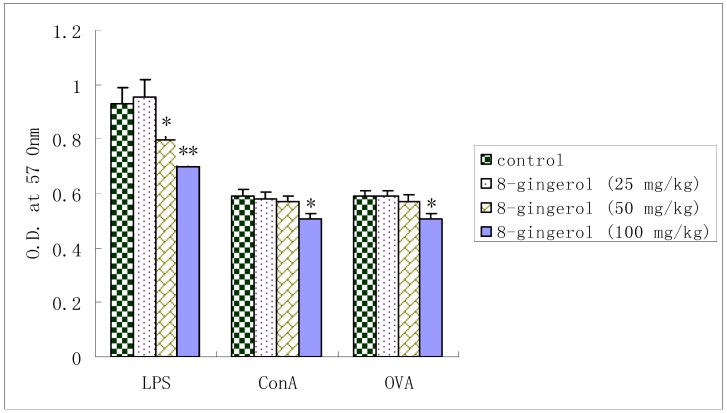
Effect of 8-gingerol on splenocyte proliferation in OVA- immunized mice.

It is generally known that Con A stimulates T cells, whereas LPS stimulates B cell proliferation. T cells are involved in cell-mediated immunity, and B cells are primarily responsible for humoral immunity. Proliferation assays showed that 8-gingerol could not only suppress the Con A- and LPS-stimulated splenocyte proliferation *in vitro* (Figure 3), but also decrease the Con A-, LPS- and OVA induced splenocyte proliferation in OVA-immunized mice (Figure 4). These results indicated that 8-gingerol could signiﬁcantly suppress the activation potential of B and T cells. This suggests that 8-gingerol could simultaneously suppress adaptive immunity and innate immunity. OVA-speciﬁc IgG, IgG1, and IgG2b antibody levels in the serum of OVA-immunized mice are shown in Figure 4.

**Figure 5:**
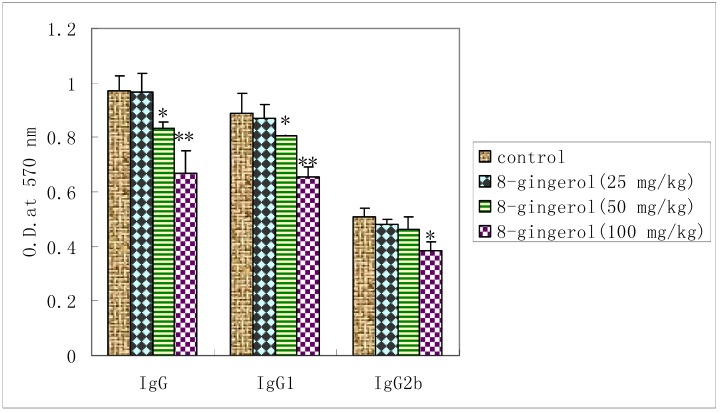
Effect of 8-gingerol on the OVA-speciﬁc serum antibody levels.

8-Gingerol signiﬁcantly decreased OVA-speciﬁc IgG and IgG1 levels at dose of 50, 100 mg/kg in mice immunized with OVA as compared with controls (*P* < 0.05 or *P* < 0.01). Meanwhile, OVA-specific IgG2b titers were also significantly decreased by 8-gingerol at dose of 100 mg/kg. These results suggest that 8-Gingerol has strong immunosuppressive effects on humoral immunity (Figure 5).

CD3^–^CD19^+^ lymphocytes were identified as B cells, while CD3^+^cells were defined as T cells. In this study, signiﬁcant suppression of CD3^-^CD19^+^ cell populations was observed after 8-gingerol treatment in a concentration-dependent manner (Table 1). These results indicate that 8-gingerol could suppress B cell activation, thereby conﬁrming the general effect of 8-gingerol on the humoral immune response. However, CD3^+^ cell populations were suppressed only at high dose 100 mg/kg of 8-gingerol, indicating that B cells are more sensitive to the effect of 8-Gingerol than T cells. These results suggested that 8-gingerol could suppress humoral and cellular immune responses in mice. The mechanism might be related to direct inhibition of sensitized T and B lymphocytes.

## 3. Experimental

### 3.1. Materials

8-Gingerol (standard compound, purity > 95%) was purchased from the National Institute for the Control of Pharmaceutical and Biological Products (Beijing, China). Ovalbumin (OVA), 3-(4,5-dimethylthiazol-2-yl)-2,5-diphenyltetrazolium bromide (MTT), concanavalin A (ConA), lipopolysaccharide (LPS), goat anti-mouse IgG, IgG1 and IgG2b peroxidase conjugates were purchased from Sigma (St. Louis, MO, USA). RPMI 1640 and FBS (Fetal bovine serum) were purchased from Gibco (Invitrogen, Carlsbad, NM, USA). Fluorescein isothiocyanate (FITC) anti-CD3, phycoerythrin (PE)-conjugated anti-CD19 antibodies were purchased from BD Pharmingen (San Diego, CA, USA). 

### 3.2. Experimental animals

Specific Pathogen Free BALB/c male mice (5 weeks old, weighing 18–22 g) were purchased from the Experimental Animal Center of Jilin University and acclimatized for 1 week before use. Mice were housed in standard conditions of temperature, humidity and light. Animal experiments were approved by Experimental Animal Center of Jilin University. All animal experiments were performed in strict accordance with the guide for the *Care and Use of Laboratory Animals* published by the US National Institutes of Health [14].

### 3.3. Cell viability assay

Cytotoxicity studies were performed by MTT assay. Splenocytes were collected from BALB/c male mice under aseptic conditions, then the lymphocyte cell suspension (2 × 10^6 ^cells/mL) was transferred into 96-well plates and incubated at 37 °C for 24 h in a humidity saturated atmosphere with containing 5% CO_2_. Cells were treated with diverse concentrations of 8-gingerol (0–100 µg/mL) for another 24 h, then 20 μL of MTT solution (5 mg/mL) were added to each well followed by 4 h incubation. The culture medium was removed and cells were lysed with 150 μL/well DMSO and followed by 10 min of shaking. The absorbance was measured at 570 nm with a microplate reader [14]. 

### 3.4. In vitro splenocyte proliferation assay

Splenocytes were collected from BALB/c male mice under aseptic conditions. The viability of splenocytes was determined by the trypan-blue dye exclusion technique. Cell viability exceeded 95%. Splenocyte proliferation was assayed. Brieﬂy, splenocytes were seeded into a 96-well ﬂat-bottom microtiter plate (Nunc) at 5 × 10^6^ cell/mL in 100 µL complete medium. Thereafter, Con A (ﬁnal concentration 5 µg/mL), LPS (ﬁnal concentration 20 ug/mL) or RPMI 1640 medium with 8-gingerol (ﬁnal concentration 10, 20, 40, 80 µg/mL) was added to a ﬁnal volume of 200 µL (four wells). The plate was incubated at 37 °C in a humidity saturated atmosphere with containing 5% CO_2_. After 44 h, 20 μL of MTT solution (5 mg/mL) was added to each well and incubated for 4 h. The culture medium was removed, 150 μL of DMSO was added to lyse the cells and followed by 10 min of shaking. The absorbance at 570 nm was measured and the stimulation index (SI) was calculated [15,16]*.*

### 3.5. Administration and immunization

The administration and immunization procedure was modiﬁed from previous studies [17,18]. In brief, six weeks old male BALB/c mice were divided into five groups consisting of eight mice each. Except negative group, all mice were immunized subcutaneously with 100 μg OVA on day 1. A boosting injection was given on day 7. On the day of the second immunization, immunized mice were treated with 8-gingerol intraperitoneally in a single dose of 25, 50 and 100 mg/kg for 7 consecutive days. Saline-treated mice were included as control. The above mentioned doses of 8-gingerol did not cause any mortality and were considered safe in this experiment. On day 14, splenocytes and serum were obtained for proliferation assays, measurement of CD3 T and CD19 B lymphocyte subsets and OVA-specific antibody levels.

### 3.6. In vivo splenocyte proliferation

Splenocytes were collected from the OVA-immunized BALB/c mice and prepared as described above, seeded into a 96-well ﬂat-bottom microtiter plate at 5 × 10^6^ cell/mL in 100 µL complete medium, thereafter Con A (ﬁnal concentration 5 µg/mL), LPS (ﬁnal concentration 20 µg/mL), OVA (ﬁnal concentration 20 µg/mL), or complete RPMI 1640 medium were added to a ﬁnal volume of 200 µL (four wells). The plates were incubated at 37 °C in a humidiﬁed atmosphere with 5% CO_2_. After 44 h, splenocyte proliferation was assayed as described before.

### 3.7. Analysis of cell surface markers by ﬂow cytometry

Spleens were collected from OVA-immunized BALB/c mice and prepared as described above. Splenocyte surface markers were detected by staining cells with PE-conjugated anti-CD19 antibody and FITC-conjugated anti-CD3 antibody. Brieﬂy, 1 × 10^6^ cells were washed twice with wash buffer (PBS containing 0.1% NaN_3_). The samples were then incubated with antibodies for 20 min at 4 °C in the dark. Each sample was resuspended in 0.5 mL ﬁxing solution (PBS containing 2% formaldehyde and 0.05% NaN_3_) and analyzed on a BD FACS Calibur ﬂow cytometer (Becton-Dickinson, San Jose, CA, USA). CellQuest software (Becton-Dickinson) was used to identify and quantify distinct populations of cells by mean ﬂuorescence intensity (MFI). A minimum of 10,000 cells were analyzed for each sample [19,20]*.*

### 3.8. Measurement of OVA-specific antibody levels by ELISA

OVA-specific IgG, IgG1 and IgG2b antibodies in serum were detected by ELISA. In brief, ELISA plates were coated overnight at 4 °C with 100 μL/well OVA solution in coating buffer (0.1 M NaHCO_3_ and 0.034 M Na_2_CO_3_; pH 9.5). The wells were washed three times with PBS containing 0.05% (v/v) Tween 20 (PBS/Tween) and then blocked with 1% FCS/PBS at 37 °C for 1 h. After three washings, 100 μL of serum samples (1:100 dilution in blocking buffer) were added to each well. The plates were covered and incubated at 37 °C for 1 h. The plates were then washed three times and goat horseradish peroxidase (HRP)-conjugated anti-IgG, IgG1 or IgG2b antibody (1:2,000 dilution in blocking buffer) was added (100 μL/well) to the wells and plates were then incubated at 37 °C for 2 h. The plates were washed again and the bound peroxidase conjugate was detected by addition of substrate solution (100 μL/well) containing 0.1 mg/mL of tetramethylbenzidine (TMB). The reaction was terminated by adding 50 μL/well of 2N H_2_SO_4_ solution and the absorbance was measured in an ELISA reader at 450 nm with a 490 nm reference. ELISA assays were performed on the same day for all samples.

### 3.9. Statistical analysis

All values were expressed as means ± standard deviations. Differences groups were assessed by Fisher’s exact test. Differences between mean values of normally distributed data were assessed by one-way ANOVA (Dunnett’s t-test) and Student’s t-test. Statistical difference was accepted at P < 0.05.

## 4. Conclusions

The data reported here firstly suggested that 8-gingerol simultaneously inhibited the humoral and cellular immune responses in mice by directly suppressing B- and T-cell activation and deserved further researches as a promising immunosuppressant. 
